# Birth preparedness and place of birth in Tandahimba district, Tanzania: what women prepare for birth, where they go to deliver, and why

**DOI:** 10.1186/s12884-016-0945-5

**Published:** 2016-07-16

**Authors:** Tara Tancred, Tanya Marchant, Claudia Hanson, Joanna Schellenberg, Fatuma Manzi

**Affiliations:** London School of Hygiene and Tropical Medicine, London, UK; Karolinska Institute, Stockholm, Sweden; Ifakara Health Institute, Dar es Salaam, Tanzania

**Keywords:** Pregnancy, Childbirth, Facility delivery, Birth preparedness, Tanzania

## Abstract

**Background:**

As making preparations for birth and health facility delivery are behaviours linked to positive maternal and newborn health outcomes, we aimed to describe what birth preparations were made, where women delivered, and why.

**Methods:**

Outcomes were tabulated using data derived from a repeated sample (continuous) quantitative household survey of women aged 13–49 who had given birth in the past year. Insights into why behaviours took place emerged from analysis of in-depth interviews (12) and birth narratives (36) with recently delivered mothers and male partners.

**Results:**

Five hundred-twenty three women participated in the survey from April 2012–November 2013. Ninety-five percent (496/523) of women made any birth preparations for their last pregnancy. Commonly prepared birth items were cotton gauze (93 %), a plastic cover to deliver on (84 %), gloves (72 %), clean clothes (70 %), and money (42 %). Qualitative data suggest that preparation of items used directly during delivery was perceived as necessary to facilitate good care and prevent disease transmission. Sixty-eight percent of women gave birth at a health facility, 30 % at home, and 2 % on the way to a health facility. Qualitative data suggested that health facility delivery was viewed positively and that women were inclined to go to a health facility because of a perception of: increased education about delivery and birth preparedness; previous health facility delivery; and better availability and accessibility of facilities in recent years. Perceived barriers: were a lack of money; absent health facility staff or poor provider attitudes; women perceiving that they were unable to go to a health facility or arrange transport on their own; or a lack of support of pregnant women from their partners.

**Conclusions:**

The majority of women made at least some birth preparations and gave birth in a health facility. Functional items needed for birth seem to be given precedence over practices like saving money. As such, maintaining education about the importance of these practices, with an emphasis on emergency preparedness, would be valuable. Alongside education delivered as part of focussed antenatal care, community-based interventions that aim to increase engagement of men in birth preparedness, and support agency among women, are recommended.

## Background

Facility delivery with a skilled attendant in a centre providing emergency obstetric care is the primary strategy to reduce maternal and newborn mortality [1–5]. Tanzania benefits from having a fairly decentralised health system in which approximately 80 % of the population lives within five kilometres of a health facility [6]. Although likely an underestimate in 2016, the most recent demographic and health survey for Tanzania in 2010 suggested that only 50 % of births occurred in a health facility [7].

Several studies have indicated that birth preparedness is associated with uptake of health facility delivery [8–11]. Recommended birth preparedness plans differ between countries, but most include: planning the location of delivery and knowing the location of the nearest health facility; identifying a birth attendant; saving money for birth-related and emergency expenses; making arrangements for transport to a health facility for birth or complications; and identification of a blood donor [8]. In 2002, Tanzania adopted a birth preparedness and complication readiness strategy as part of focussed antenatal care, with the overall goal of increasing facility births [12]. The strategy emphasised knowing the expected delivery date; identifying a place of birth; identifying someone to care for the woman’s family in her absence; preparing essential items needed for a clean birth; identifying at least two blood donors; preparing funds for transportation; identifying a decision-making family member to accompany a woman during labour; and the importance of delivering in a health facility [12–14]. This new approach also suggested movement away from a “risk approach” strategy that placed emphasis on facility delivery for women with high-risk pregnancies, which is now emphasised for all women [15].

Although the intended connection between birth preparedness and health facility delivery is clear, the two are not often reported together [9, 16]. In the context of a quality improvement project to improve maternal and newborn health in Tandahimba district of southern Tanzania (the Expanded Quality Management Using Information Power (EQUIP) project) [17–19], we investigated how many women made birth preparations, what they prepared, and what their place of delivery was. We then used qualitative data from in-depth interviews and birth narratives to explain why birth preparations were made and what determined place of delivery to further explore the relationship between the two practices.

## Methods

Results were derived from a continuous quantitative household survey conducted April 2012–November 2013, and qualitative in-depth interviews and birth narratives conducted in 2013.

### Study setting

The study setting has been described in more detail elsewhere [17]. Tandahimba is a predominantly rural district with a population of 227 500 [20]. It has one district hospital, three health centres, and 30 dispensaries. As of 2010, maternal and newborn mortality rates in Tandahimba were higher than national averages at 712 deaths per 100 000 live births and 31 deaths per 1000 live births respectively [7, 21]. The majority of inhabitants are from the Makonde ethnic group, are Muslim, and their primary economic activity is farming, particularly of cashew nuts [22, 23].

### Quantitative data collection

Quantitative data were collected as part of a continuous cross-sectional modular household survey (see Marchant et al.) [18]. Briefly, the probability sampling scheme for this survey was designed to be representative at the district level, with six rounds of data being collected from November 2011–April 2014. In each round, approximately 2300 households were surveyed and all consenting resident women aged 13–49 years were interviewed. Overall 89 % of eligible women participated in the survey. In the women’s module, participants with a recent live birth (a birth within the past 12 months) were identified using pregnancy histories, then asked about place of delivery for that birth, whether they had made birth preparations, and if so, to report which items they had prepared. Such that participants would be responding around pregnancy or childbirth in approximately the same timeframe, data are presented from April 2012–November 2013 only to allow for temporal overlap with the period referenced by women during qualitative data collection.

### Qualitative data collection

Qualitative data were collected from 12 semi-structured in-depth interviews with mothers who had recently given birth, and 23 birth narratives with recently delivered mothers and 13 men whose partners had recently given birth. No participants refused to partake. Birth narratives were considerably more open than in-depth interviews, leaving opportunities for participants to share their narrative around their or their partner’s experiences in pregnancy and childbirth. However, interviewers probed as necessary to ascertain greater detail or explanation. All in-depth interviews and birth narratives were carried out in Swahili. Participants were selected to be as diverse as possible (according to age, number of children, general socioeconomic status, place of delivery, whether a caesarean section was carried out, and if twins had been born for themselves or their partners).

### Analysis

Stata 13 was used to generate descriptive statistics for survey respondents with regard to age, marital status, religion, and birth preparation and place of delivery outcomes. Percentages were generated using the *svy* command to account for the clustered survey design, and statistical evidence of association between birth preparedness and place of delivery was determined using a weighted Pearson’s chi-squared test.

Qualitative data were coded line-by-line using NVivo 10 software to generate themes around participant rationale for making birth preparations (or not), or delivering in a health facility (or not). All qualitative in-depth interviews and birth narratives were analysed through constant comparison, and interview or narrative guides were adjusted to probe further into emerging themes or to further explore divergent cases. Data were collected and analysed until theoretical saturation had been reached. Emergent themes are reflected in the results through representative quotations.

## Results

### Participant characteristics

Five hundred and twenty-three women aged 13–49 who had given birth in the previous 12 months at the time of the survey (April 2012–November 2013) participated in the continuous survey (Table 1). The majority of these women were married and Muslim, with 70 % of respondents aged 20–39 years.

**Table 1:** Continuous survey (April 2012–November 2013) participant characteristics with a birth in the previous year

Participant characteristics	Number	Percent
Age		
13–19	93	18
20–29	211	40
30–39	158	30
40–49	61	12
Total	523	100
Marital status		
Currently married	408	78
Previously married	58	11
Never married	41	8
Unmarried but living with partner	16	3
Total	523	100
Religious background		
Christian	8	2
Muslim	515	98
Total	523	100

Among participants of in-depth interviews and birth narratives, mothers’ ages ranged from 16 to 44 years, with an average age of 27. Mothers’ parity ranged from one-to-six, and 12 women out of 35 had given birth at home. Fathers’ ages ranged from 21 to 60 years, with an average age of 36. The number of children for each father ranged from one-to-eight, with four out of 13 of their partners delivering at home. All participants were married and had a similar age distribution to surveyed women. Please see Tancred et al. for more detail about participants [19].

### Birth Preparedness

#### Quantitative findings

In the continuous survey, 95 % (496/523, 95 % CI 92–97 %) of women reported making birth preparations for the last live birth that they had in the 12 months prior to the survey. When asked to list what they had prepared, women reported some items more commonly than others (Table 2). Of the recommended items for birth preparedness, cotton gauze, a cover to deliver on, gloves, and clean clothes were prepared by almost 70 % or more of all respondents. Money was prepared by 42 % of respondents, and other recommended items needed during labour and delivery like a razor, a basin, and soap were cited by 10–20 % of participants. Arrangement of transport and identification of a health facility for delivery was stated by only 2 % or less of respondents.

**Table 2:** Birth preparedness among survey respondents who had given birth in the previous year

Items prepared	n/496	Percent	95 % CI
Cotton gauze	460	93	90–95
Cover to deliver on	418	84	81–87
Gloves	359	72	67–77
Clean clothes	267^a^	70	65–74
Money	206	42	37–46
Razor	86	17	14–21
Basin	64	13	10–16
Soap	56	11	8–15
Cord clamps or thread	52	10	8–13
Bucket	51	10	8–13
Uterotonic drugs	26	5	3–8
Transport	9	2	1–3
Identification of facility for delivery	3	1	0–2

^a^ *N* = 384 due to missing values

#### Qualitative findings

##### Birth items prepared

All of the items in Table 2 were also cited during in-depth interviews and birth narratives, with some insights as to why they were prepared. For example, a bucket for carrying water or disposal of placenta, a basin for washing clothes, thread or a cord clamp for tying the umbilical cord, a clean razor for cutting the umbilical cord, and soap, both for washing clothing, or for washing the mother. Preparation of uterotonic drugs like oxytocin to be used by a skilled birth attendant to induce labour or to prevent and treat post-partum bleeding and a syringe or needle to administer them was also indicated by participants. The amount of money prepared stated by participants in in-depth interviews ranged from as low as 12 000 Tanzanian shillings (~7.5 USD) to 100 000 Tanzanian shillings (~64 USD). Arrangement of transport was regularly mentioned as something that was done when a woman went into labour, rather than a consideration that was made ahead of time when other birth preparations were made.

##### Rationale for making birth preparations: use in delivery and influence on quality of care

Qualitative data provided some insight as to why some items were prepared more commonly than others. All participants made at least some birth preparations. As reflected in Table 2, items used directly in birth were perceived to be of particular importance. Further to their immediate use during delivery, having these items was linked by participants to the care that would be received in a health facility; together, these were the primary motivators for women to make preparations. It was even stated by a few respondents that not making preparations might push a women to have a home birth for fear of refusal at the health facility.

*“In the hospital during service, if they ask you to bring gloves, you give them, bring a bucket, I give them, so services go well. [Interviewer: What could have happened if you did not have those items?] They could have refused to help me in the hospital.” (Mother, 38)*

An additional motivator for women was that they felt that these items were important to help in the prevention of infectious disease transmission. Having your own plastic sheet to deliver on, gloves, and a clean razor were seen to be of particular importance for this reason. Many referred to “homa kubwa” (the “big fever”), referring to HIV, and suggested that there are more diseases “nowadays” that women need to be protected against than in the past. As such, preparation of birth items was seen as essential to prevent the transmission of infections such as HIV.

*“You know nowadays there a lot of diseases, so if we use the same equipment there is a possibility of disease transmission.” (Mother, 44)*

##### Understanding what to prepare: education and inclusion of men

Irrespective of parity, it was commonly stated that the education around birth preparedness that was given by EQUIP volunteers to mothers and fathers in their homes was useful in helping women to know exactly what to prepare. Women who had previously given birth indicated that for their past births, many of the functional items used in delivery such as gloves, a razor to cut the cord, a plastic sheet for laying on the bed, and others, were typically found in the hospital. Now, the expectation was for women to bring these items with them to the health facility.

*“[Without encouragement from the EQUIP volunteers] I could not have prepared myself because during the previous pregnancies you find all those things in the hospital. I couldn’t have known what to prepare—probably I would have carried a piece of khanga [cloth], thinking that all the services are available at the hospital.” (Mother, 39)*

Data from birth narratives and in-depth interviews suggested that men were typically charged with the responsibility of purchasing birth items, or conversely, giving money to their partners for them to buy the items. As such, men played a key role in ensuring birth preparations were made. It was perceived that, where birth preparations were not made, it was failure of the male partner, either because he was no longer in the pregnant woman’s life, or because he had failed to purchase the items due to financial constraints or lack of will. Making birth preparations was seen to be a particularly difficult undertaking for single pregnant women.

*“It is possible there is a person whom you depend on, and [he] is poor. He doesn’t have money, like your parents [who are also poor], and the one who made you pregnant has rejected you, and if the parent has little capacity, that equipment [for birth] won’t be available.” (Mother, 19)*

##### Timing of birth preparations

Finally, although EQUIP volunteers and health facility staff stressed the importance of starting to prepare early in a pregnancy, some participants held the view that items could be purchased at the health facility if preparations were not complete by the time of delivery. Furthermore, some had been told to replace prepared items while being at the facility, so they perceived early preparation to be futile.

*“I planned to buy those things when I got money, but I felt labour pain without finishing doing delivery preparations, so I went to the hospital and got all the needed things there.” (Mother, 24)*

*“I won’t prepare, rather, I will save money. I will just buy things at the hospital in case there are things that I will be asked to buy. I already know all things are sold there.” (Father, 32)*

### Place of delivery

#### Quantitative findings

Table 3 below shows place of delivery results from the continuous household survey and highlights what percentage of women delivering at each place also reported making birth preparations. Overall, 68 % of births took place in a health facility and only 30 % at home. Among all facility births, 99 % of mothers made any birth preparations, compared to only 86 % of mothers delivering at home (chi-squared test *p*-value <0.001).

**Table 3:** Place of delivery and birth preparations made among survey respondents

Place of delivery	n/N	Percent	95 % CI	Birth preparations made (n/N)	Percent	95 % CI
Hospital	164/526	31	25–38	161/163	99	95–100
Health Centre	50/526	10	6–15	50/50	100	100
Dispensary	144/156	27	22–33	142/144	99	94–100
Home	156/526	30	25–35	132/154	86	78–91
Other	12/156	2	1–4	11/12	92	56–99

#### Qualitative findings

##### Rationale for health facility delivery

Health facility delivery was viewed very positively. The key perceived benefit of health facility delivery was that safety for the mother and the newborn were ensured. The most commonly cited reasons as to why health facility delivery had increased included:

- increased education of mothers and fathers about maternal and newborn health, both received at health facilities during antenatal care, but also from village volunteers like those from EQUIP;
  *“I received education for my second child. Now they don’t allow anyone to deliver at home. Most of us now go to the hospital for delivery.” (Mother, 25)*
- special efforts being made to sensitise women who are young or primiparous or have had five or more children about the necessity of them delivering in a hospital due to their increased risk of complications;
  *“They said that this is the fifth pregnancy, once the person reaches the fifth pregnancy they should go to the hospital.” (Mother, 35)*
- women having previously experienced complications and therefore understanding the importance of health facility delivery;
  *“[For the first pregnancy] the baby was too big, so they enlarged her birth canal [gave her an episiotomy]—that couldn’t be done at home … [Because] I saw that the first pregnancy had developed complications…I told [my wife] to prepare herself to go to the hospital.” (Father, 35)*
- women generally having positive experiences at the health facility and choosing to return for future births;
  *“I have seen great success in my first pregnancy; I didn’t face any problems…they followed up and I did listen to them…I have seen its importance.” (Mother, 19)*
- the prohibition of homebirths in some villages often through the use of fines for mothers or for traditional birth attendants who may be assisting them—established by village leaders or by volunteers like those in EQUIP as part of their strategies—or the refusal of services by local health facility staff (see Tancred et al. for more detail on this point [19]);
  *“But if you deliver at home [then] at the time you go to facility, they refuse to attend you, other staff may even refuse to give you a card.” (Mother, 19)*
- an increased number of facilities and more reliable modes of transportation, namely motorbikes.
  *“[Health facilities] were few, and we used to go for long distances and there was no reliable transport. People used to carry pregnant women on a bicycle or in a basket and take them to hospital, but now if labour pains start they take them faster using motorbikes.” (Mother, 38)*

##### Reasons for home births

Women who had home births said that they had made birth preparations with the intention to deliver in a health facility. They commonly reported that the home birth occurred because they were alone in the house with her partner working or travelling elsewhere. One consequence of being alone was that a woman may have failed to get transport to a health facility, as men typically took on the responsibility of arranging and paying for transport. Interestingly, such was the case even if these women had been left money by their partners, suggesting a potential need for female agency in the absence of others—husbands or other family members—who would make the decision to seek care. Participants did not refer to emergency preparedness for the situation when a woman might be alone and starting labour.

*“I was alone…My husband was not around and my children were at school, it was around two…on the way back from school, my child went to tell his father in order to find transport…When my husband arrived with transport I had already delivered.” (Mother, 29)*

*“I was alone…I didn’t intend to [give birth at home], I didn’t know [I was in labour] and I stayed for a long time…My husband left enough money—when he travelled he left me one hundred thousand shillings [~64 USD].” (Mother, 24)*

If her partner was present and a woman still gave birth at home, it was typically due to no health facility staff being present, which was particularly true among women accessing dispensaries. Poor provider attitudes were also seen to discourage women from attending the health facility.

*“The labour pain started and we sent her there [to the dispensary], but there was no one to attend her … The problem is, there are only two staff, and if they [leave], this facility remains with no one.” (Father, 40)*

*“The nurse just throws the patient on the bed, until the one who has come to look after the patient follows the nurse and asks her to go and look at her patient but she doesn’t and she says, ‘I feel sleepy, I am going to sleep’. So she goes to call the traditional birth attendant in the village to help with the birth [instead].” (Mother, 35)*

Otherwise, childbirth at home was reported to be an accident or something that occurred in an urgent and unexpected situation. Less commonly, respondents discussed a lack of knowledge on the mother’s behalf, or financial struggles that would prevent a woman from being able to get to a health facility at all. As for both the long-term preparation of money and items needed for delivery, and the short-term arrangement of transportation, the need for a present male in order to make decisions was key, and as such, the absence of a male partner—either a temporary absence, or if a woman was no longer with her partner at all—was also regularly noted as a reason that may cause women to deliver from home.

*“There are changes because nowadays there is the use of services professionally, and mothers are educated and they go to deliver at the health facility. Nowadays to give birth at home is an emergency.” (Mother, 39)*

*“Others might have no money. Another problem is she might have no one to take her to the health facility. Some are single mothers. Some…women are rejected, while others do not have relatives to help them.” (Mother, 30)*

## Discussion

As seen in other studies from Tanzania, birth preparedness was carried out among the vast majority of pregnant women [24, 25]. Qualitative results highlighted that items that would be used directly in delivery were perceived to be of the greatest importance. The perception that having these items would ensure that appropriate care was received and would also be instrumental in minimising infectious disease transmission was widely held. Health facility delivery was an increasingly popular behaviour, with only 30 % of births being carried out at home. As has been found in other settings, increased education to parents about maternal and newborn health—including that received from village volunteers like those of EQUIP [26–28], positive past experiences at health facilities [29–32], prohibition of homebirths in some villages, and increased accessibility of health facilities were all perceived to be important contributors to this decrease in homebirths [28, 30, 33]. Qualitative data highlighted that in the rare instances where birth preparedness or health facility delivery were not done, the primary causes were: delaying to travel to a health facility; a lack of health facility staff or poor provider attitudes; financial barriers; and a lack of male involvement [16, 28, 33–37]. Finally, the link between birth preparedness and health facility delivery in this setting was highlighted by our finding that, although 86 % of women who gave birth at home made at least some preparations, they were significantly less likely to have done so than those delivering at a facility (99 %). This relationship should continue to be explored across settings.

Given the link between birth preparedness and health facility delivery, there is an added value of having community-based volunteers who are in a position to reiterate messaging around both within a family context, and to follow-up to ensure birth preparations were being made. However, there has been a failure to take up some aspects of birth preparedness as suggested in Tanzanian policy, including the identification of a blood donor, which was not stated by any respondents. There also appears to be a need to underline the importance of making preparations from early in pregnancy, and emergency preparedness around getting to a health facility in the event of unexpected or early labour. The attitude expressed by some that the functional items to be used during birth could simply be bought at the health facility might also lead to a delay in preparedness. If an insufficient amount of money has been saved, those items might not be purchased at all, which, as participants suggested, might inhibit care-seeking during delivery. The perceived refusal of services to women who were not prepared for birth should be addressed through supportive supervision and provider education.

It has been well documented that in Sub-Saharan Africa, as in other settings, males strongly influence payment for birth items, transportation to health facilities, and decision-making around care-seeking practices [29, 38–44]. Despite Tandahimba district falling within an area of Tanzania that is matrilineal [45], qualitative data suggested that women still lacked decision-making capacity. The implications of these norms are twofold: first, women need to have increasingly more agency in terms of decision-making, especially when her partner may not be present, and second, men need to be educated about pregnancy and childbirth to the greatest extent possible. Education of males, often through attendance of antenatal care with their partners, has been found to be an important predictor of involvement in birth preparedness and childbirth [41, 46, 47]. A benefit of community-based initiatives is that they are positioned to support the engagement of men in pregnancy and childbirth [48]. The ongoing encouragement of male involvement in antenatal care may be a particularly useful strategy to provide a platform for education. Future research on the role of males and the decision-making capacity of women around birth preparedness and facility delivery in this context would be valuable.

### Limitations

The household survey was carried out throughout the entire district of Tandahimba, but qualitative data were only collected from one division. The question around birth preparedness was open, with women encouraged to state anything they had prepared rather than being asked to respond to a structured list. Given that almost 70 % of births occurred at a facility, this method may have resulted in an underestimate of identification of a facility, saving money, and arrangement of transport, which other studies have reported to occur more frequently [24, 25]. Finally, as there is a very strong understanding that health facility delivery and birth preparedness are favourable behaviours, there is the possibility that data were influenced by responder bias, with participants responding more positively about both practices than actually occurred.

## Conclusions

This study highlighted that the majority of women make at least some birth preparations and give birth in health facilities. Women seemed to place importance on functional items needed for delivery rather than on arranging transport or identifying a health facility, and did not always appreciate the importance of making birth preparations early. As such, there is a need to emphasise emergency preparedness in education to women and their partners during antenatal care. Furthermore, to address some barriers to making preparations or delivering in a health facility, it would also be beneficial to continue to encourage increased male engagement in pregnancy and childbirth as well as greater female agency around both. Community-based interventions may be well poised to work toward these aims.

## Abbreviations

EQUIP, Expanded Quality Management Using Information Power
